# Diet in Parkinson's Disease: Critical Role for the Microbiome

**DOI:** 10.3389/fneur.2019.01245

**Published:** 2019-12-10

**Authors:** Aeja Jackson, Christopher B. Forsyth, Maliha Shaikh, Robin M. Voigt, Phillip A. Engen, Vivian Ramirez, Ali Keshavarzian

**Affiliations:** ^1^Division of Digestive Diseases, Department of Internal Medicine, Rush University Medical Center, Chicago, IL, United States; ^2^Graduate College of Rush University, Chicago, IL, United States

**Keywords:** Parkinson's disease, diet, microbiome, SCFA, LPS, intestinal hyperpermeability, vagus nerve, GLP-1

## Abstract

**Background:** Parkinson's disease (PD) is the most common movement disorder affecting up to 1% of the population above the age of 60 and 4–5% of those above the age of 85. Little progress has been made on efforts to prevent disease development or halt disease progression. Diet has emerged as a potential factor that may prevent the development or slow the progression of PD. In this review, we discuss evidence for a role for the intestinal microbiome in PD and how diet-associated changes in the microbiome may be a viable approach to prevent or modify disease progression.

**Methods:** We reviewed studies demonstrating that dietary components/foods were related to risk for PD. We reviewed evidence for the dysregulated intestinal microbiome in PD patients including abnormal shifts in the intestinal microbiota composition (i.e., dysbiosis) characterized by a loss of short chain fatty acid (SCFA) bacteria and increased lipopolysaccharide (LPS) bacteria. We also examined several candidate mechanisms by which the microbiota can influence PD including the NLRP3 inflammasome, insulin resistance, mitochondrial function, vagal nerve signaling.

**Results:** The PD-associated microbiome is associated with decreased production of SCFA and increased LPS and it is believed that these changes may contribute to the development or exacerbation of PD. Diet robustly impacts the intestinal microbiome and the Western diet is associated with increased risk for PD whereas the Mediterranean diet (including high intake of dietary fiber) decreases PD risk. Mechanistically this may be the consequence of changes in the relative abundance of SCFA-producing or LPS-containing bacteria in the intestinal microbiome with effects on intestinal barrier function, endotoxemia (i.e., systemic LPS), NLRP3 inflammasome activation, insulin resistance, and mitochondrial dysfunction, and the production of factors such as glucagon like peptide 1 (GLP-1) and brain derived neurotrophic factor (BDNF) as well as intestinal gluconeogenesis.

**Conclusions:** This review summarizes a model of microbiota-gut-brain-axis regulation of neuroinflammation in PD including several new mechanisms. We conclude with the need for clinical trials in PD patients to test this model for beneficial effects of Mediterranean based high fiber diets.

## Introduction

Parkinson's disease (PD) is recognized as the second most common neurodegenerative disease of aging after Alzheimer's disease (AD) and the most common movement disorder, affecting up to 1% of the population above the age of 60 and 4–5% of those above the age of 85 (1, 2). While there are treatments that minimize symptoms of PD, little progress has been made on efforts to halt disease progression (3). Less than 10% of PD is associated with specific genetic changes, which means that the search is on for environmental risk factors for PD (3, 4). Diet is one such environmental factor that has emerged as a potential factor that can promote the development or exacerbate the progression of PD (5–7). In this review, we will discuss evidence for the diet involvement in PD development, discuss the mechanisms by which the diet-mediated effects on the microbiome may influence PD, and also discuss how dietary interventions may be used to prevent or treat PD.

## Diet in Parkinson's Disease

**Figure 1:**
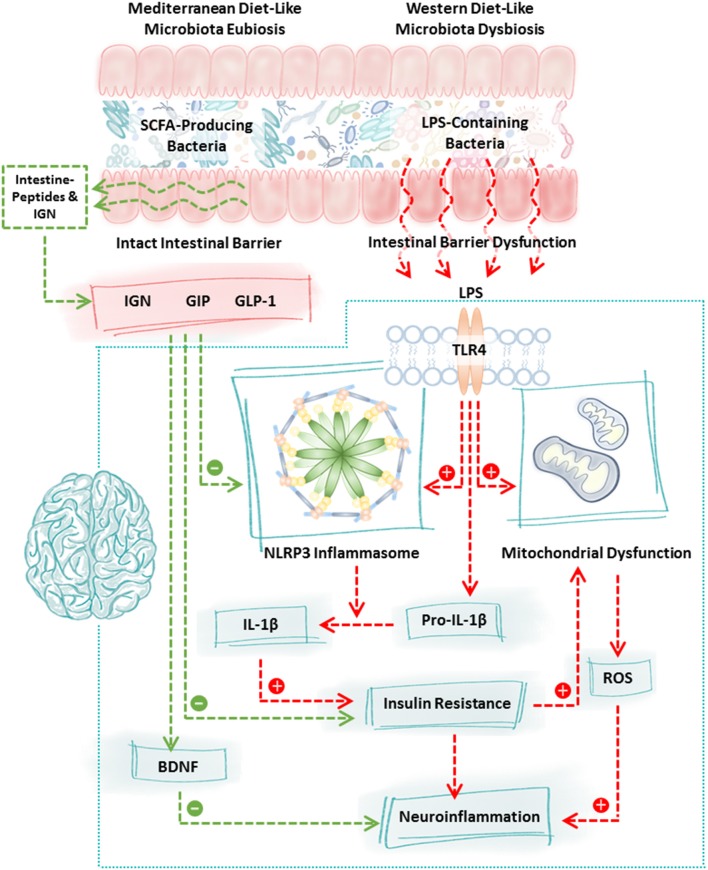
Mechanisms of communication between the intestinal microbiota and the brain. Diet robustly impacts the intestinal microbiota. Consumption of a Western diet (or components of the Western diet) promotes the growth of LPS-containing bacteria and reduces the abundance of SCFA-producing bacteria whereas consumption of a Mediterranean diet (or components of the Mediterranean diet) promotes the growth of SCFA-producing bacteria and reduces LPS-containing bacteria. This shift is highly significant because LPS-containing bacteria are pro-inflammatory, they disrupt intestinal barrier integrity and LPS binding to TLR4 stimulates a cascade of events including NLRP3 inflammasome activation, mitochondrial dysfunction, and insulin resistance culminating in neuroinflammation and neurodegeneration. In contrast, increased production of SCFA due to consumption of the Mediterranean diet (or components of the Mediterranean diet) fortifies the intestinal barrier, stimulates the intestinal L-cell production of GLP-1 and GIP which inhibits NLRP3 inflammasome activation and normalizes insulin resistance. SCFA also stimulate intestinal epithelial cell IGN and together with GLP-1/GIP stimulate the vagus nerve and brain BDNF which has numerous beneficial effects on the brain and which improves neuron insulin resistance all of which function to promote neuronal health. Characteristic features of the PD microbiome are similar to those observed following consumption of the Western diet (low SCFA-producing bacteria, high LPS-containing bacteria); therefore, dietary interventions such as the Mediterranean diet (or components of the Mediterranean diet) may be a viable approach to blunt neuroinflammation and improve neuronal function in PD. BDNF, brain derived neurotrophic factor; GIP, gastrointestinal peptide; GLP-1, glucagon like peptide 1; IGN, intestinal gluconeogenesis; IL-1β, interleukin 1 beta; LPS, lipopolysaccharide; NLRP3, nucleotide-binding domain, leucine-rich-containing family, pyrin domain-containing-3; ROS, reactive oxygen species; SCFA, short chain fatty acids; TLR4, toll-like receptor 4.

There is a growing body of epidemiological evidence to support that diet impacts (positively or negatively) the development of neurodegenerative diseases such as PD. The Western diet is among the greatest risk factors for developing neurodegenerative diseases such as PD (8, 9). The Western diet is characterized by high caloric intake of energy dense foods, high in saturated and omega-6 (ω6) fatty acids, refined sugars, excessive salt intake, and low consumption of omega-3 (ω3) fatty acids and fiber (10–12). Studies of PD patients support total caloric intake of macronutrient and micronutrient correlate with symptom severity, with higher caloric intake associated with worse PD-related symptoms (13). Consumption of high quantities of animal saturated fat has been widely reported to be associated with increased risk of developing PD (14, 15). Foods associated with more rapid PD progression include canned fruits and vegetables, soda, fried foods, beef, ice cream, and cheese (all characteristic of the Western diet) (Figure 1) (9).

On the flip side, a “healthy” diet is associated with beneficial effects relative to PD (6). Adherence to the Mediterranean diet is associated with lower probability of developing PD (16). Specific components of the Mediterranean diet are particularly associated with these beneficial effects such as fresh vegetables, fresh fruit, nuts, seeds, non-fried fish, olive oil, wine, coconut oil, fresh herbs, and spices. Consumption of flavonoid-rich foods (tea, berry fruits, apples, red wine, and orange/orange juice) are also associated with a lower risk of developing PD (17). Polyunsaturated fatty acids (**PUFA**) are also inversely correlated with PD development (higher consumption of ω3 fatty acids is associated with reduced PD risk) demonstrating the influence of dietary fat intake on the brain (18, 19).

Diet can impact the body through multiple different mechanisms including direct effects of dietary components (e.g., vitamins, fats) on the body, but diet may modulate the development and/or progression of PD indirectly through effects on the intestinal microbiome (6, 20, 21). Indeed, diet is perhaps the single greatest factor determining the structure and metabolic function of the intestinal microbiota (22–25).

Coffee and caffeine in the diet have also been consistently associated with decreased risk of PD. Several key early studies showed a significant dose dependent decrease in risk for PD with increasing coffee consumption and for smoking as well (26–28). Recent studies have confirmed a decreased risk for PD in men and women with increasing caffeine consumption (29, 30). Both caffeine (coffee) and nicotine (smoking) have been shown to ameliorate disease in MPTP rodent models of PD (31, 32). In addition, coffee has recently been shown to contain chlorogenic acid that inhibits the NLRP3 inflammasome (33) and polyphenols that have been shown to be neuroprotective (34, 35) as well as promote healthy microbiome metabolism (36). Significantly, two recent reviews that discussed the beneficial effects of caffeine in reduced PD risk both propose a role for the microbiome (37, 38).

With regard to alcohol consumption and PD, there does not seem to be a clear conclusion. Two early large prospective studies showed no effect of moderate alcohol consumption and PD incidence (39, 40). However, another systematic review found a protective inverse relationship between alcohol use and PD (41). Another study found that heavy alcohol use was associated with decreased risk for PD (42). A recent review of all alcohol-PD studies concluded that prospective studies tended to find no association between alcohol use and PD with 2 studies finding an increased risk with moderate alcohol use and PD (43). However, the case-control studies were more likely to find a protective effect (43). Alcohol has also been shown to promote intestinal leakiness and microbiome effects (44–46). Thus, it appears there is no definitive view for the effects of alcohol consumption and risk for PD.

Consumption of dairy products is another area of diet that has evidence related to PD risk. Several studies have supported the view that high consumption of milk and possibly dairy products in general are associated with increased risk for PD (47–49). A diet study in Greece also found association of dairy and milk consumption with PD (50). Other more recent studies also supported association of dairy product consumption and increased PD risk (51, 52). A study in Hawaii found greater than two glasses of milk per day was associated with decreased neural density in the SN at autopsy (53). One proposed cause for these associations has been pesticides in the milk, but there is no data to support this. An intriguing recent study implicates microbiome bacteriophages, especially associated with *Lactococcus* bacteria in dairy products, as possible negative modulators of the bacterial gut microbiome in PD (54). However, a recent position paper on dairy products and PD risk concluded that overall the evidence did not warrant alarming the public to avoid dairy products (55).

There is considerable evidence that dietary or environmental exposure to neurotoxins such as rotenone and paraquat, maneb, and related neurotoxins such as MPTP can promote Parkinson's-like neurodegeneration (56, 57). All of these neurotoxins target the mitochondria and there is longstanding evidence that mitochondria dysfunction is critical in PD development (58, 59). Dysfunctional mitochondria activate the NLRP3 inflammasome (60). Both the herbicide paraquat and antifungal maneb have been linked to PD (56). Rotenone, a broad based pesticide, is currently used in animal models of PD (61, 62). MPTP, which also targets the mitochondria like the other neurotoxins listed, is also widely used as a model for PD (63, 64). There is a large body of epidemiological and experimental evidence for increased risk of PD due to environmental and dietary exposure to these neurotoxins (63–66). An early study found that exposure to pesticides resulted in a 70% increased risk for PD (67). These neurotoxins have been shown to cause Parkinsonian symptoms and SN neurodegeneration when injected systemically or directly into the striatum (62, 64). However, the effects of these environmental toxins on the microbiome has not been studied in depth. Significantly, in a PD mouse model of oral gavage administered rotenone, marked changes in the microbiome correlated with disease markers and TLR4 expression in the intestine and SN neuron loss (68, 69). Studies by this group also showed that a uridine and fish oil diet could ameliorate PD symptoms in these mice (61). In another rodent study using rotenone IP injection, changes were also found in the intestinal microbiome similar to those in PD patients (70). These studies support the model that both oral and systemic injection of these neurotoxins/pesticides can affect the microbiome. Another recent study showed that the pesticide diazinon could modulate the microbiome community in mice (71). Thus, the effects of these neurotoxins on the intestinal microbiome appears to be an important area for future study.

Recently the possibility of α-Syn in diet has become a focus of potential causes of PD (72). α-Syn is a 140 AA protein found in the brain as well as in lesser amounts in heart, muscle and other tissues and dairy products (72, 73). The function of α-Syn is unknown but hallmark inclusions known as Lewy pathology are found in neurons of the SN in PD and it is believed to play a role in PD (74). α-Syn aggregates can take many forms but it appears as though the fibrillar (PFF) form may be the most pathogenic in the brain because injection of this form can cause PD symptoms and pathology (75–77). Several studies support a possible spread of α-Syn with prion like properties and mutations in the α-Syn gene are associated with familial PD (78, 79). Recent experiments show injection of α-Syn PFF in the stomach or intestine traffic to the brain via the vagus nerve in rodents (80, 81). If α-synuclein spreads via a prion-like mechanism, then one question becomes, what are the origins of this prion-like species? One source could be meat products (72). First, it should be noted that no study has quantified the amounts of intact α-Syn in the stool. It may be that it is degraded by digestive functions and not available for uptake or absorption by the intestines. If it does remain intact, one possibility is α-Syn uptake by gut M cells. M cell depletion prevents oral prion infectivity (82). Also T cells in the gut and dendritic cells expressing LAG3 could bind α-Syn and promote its spread (83). Leaky gut could also be a mechanism for α-Syn translocation to the systemic circulation (84). Overall, research to date has yet to directly test the contribution of dietary α-synuclein to the mechanism of initiation and progression of PD (72). However, α-Syn is found in beef, pork, chicken, and fish and many people regularly consume these meat and dairy products, but only a small fraction of the general population will develop PD. Therefore, it is unlikely that eating meat products that contain α-Syn is an independent cause of PD (72). Nonetheless, future studies tracking α-Syn in the diet systemically as well as in the intestinal tract could provide new insights to a role for this key PD protein as a potential dietary risk factor.

## Diet and the PD Microbiome

The human gastrointestinal tract (GIT) harbors trillions of microorganisms collectively referred to as the microbiome (24, 85, 86). We have a symbiotic relationship with the microbiota (the bacterial component of the microbiome). We provide them with an environment (the GIT) and food and they provide us with a myriad of benefits. The microbiota helps ward off harmful microorganisms (competitive exclusion), regulate immunity, and produce substances such as vitamins, secondary bile acids, and short chain fatty acids (SCFA) (24). For example, dietary fiber is used as a food source by the intestinal microbiota. Dietary fiber is a general term for consumed plant-based complex carbohydrates that are largely not digested by mammalian enzymes in the small intestine and consequently cannot be absorbed. However, they are available to be used as a food source by the intestinal (colonic) microbiota (87). Colonic bacterial fermentation of these dietary fibers generates metabolic byproducts and especially important are SCFA (10, 87–89). In contrast to these beneficial commensal bacteria, there are also pathogenic bacteria (pathobionts) that can cause GIT dysfunction (intestinal barrier dysfunction) and inflammation in the intestinal mucosa, systemic circulation, and even in the brain (10, 90). Thus, the balance of microbiota influences not only the GIT, but also organs throughout the body including the brain (91).

Although no two human microbiota communities are identical (influenced by lifestyle factors like diet, exercise, and genetics), recent studies in the last 10 years have shown people with certain diseases tend to share similar characteristic microbiota features (24, 92). An abnormal microbiome (so called “dysbiosis”) is associated with many human diseases such as obesity/metabolic syndrome, inflammatory bowel disease (IBD) and other chronic inflammatory diseases as well as in PD (90, 93, 94). The intestinal microbiota has become a major focus of PD studies (95, 96). Initial studies by Scheperjans et al. (97) and Keshavarzian et al. (98) reported abnormal intestinal microbiota composition (dysbiosis) in PD patients. Subsequently, 15 additional studies from the USA, Europe and Asia have also demonstrated dysbiosis in PD patients (Supplementary Table 1) (95, 96). As detailed in Supplementary Table 1, the PD patient's microbiota composition alterations are not identical in all of these studies. This is not surprising and should be expected because of the significant intra- and inter-individual variability discovered in the microbiota composition of healthy control subjects (99, 100) and other diseases, where intestinal dysbiosis has been reported (90, 92). Environmental factors, especially diet, can markedly affect microbiota community structure and composition, and thus it is expected that the intestinal microbiota in patients from the USA should be different from those living in Europe or Asia (21, 101). In fact, the intestinal microbiota was found to be significantly different in individuals living in different communities in the city of Chicago, Illinois, USA (102). The important key finding is that patients with PD have abnormal intestinal microbiota communities (“dysbiosis”) regardless of where they live and also the PD microbiota community appears abnormal still after 2 years of follow up (103, 104). The majority of PD human studies employed bacterial 16S ribosomal RNA (rDNA amplicons) sequencing to different variable regions to identify bacteria in feces (majority of studies), colonic sigmoid mucosa (98), nasal wash (105) or nasal swab and oral (106, 107) samples. Three studies used targeted quantitative PCR, while one study utilized metagenomics shotgun sequencing. Regardless of sequence technique or bioinformatics methodologies, the overall common discovery indicated dysbiotic bacterial profiles, which suggested putative pro-inflammatory bacteria were more abundant and putative beneficial bacteria were less abundant in PD patients.

Parkinson's disease subjects demonstrated significantly altered intestinal microbial compositions in comparison to healthy controls with some overall trends worthy of comment. Briefly these include PD subjects to exhibit: increased relative abundance of genera *Akkermansia* (7 studies) (98, 105, 108–112), *Bifidobacterium* (5 studies) (103, 108, 110, 113, 114), and *Lactobacillus* (7 studies) (97, 103, 110, 111, 113, 115, 116); decreased abundance of genus *Prevotella* (7 studies) (97, 103, 104, 108, 109, 113, 117) and the family Lachnospiraceae (6 studies) (98, 103, 110, 111, 114, 117) along with its lower taxonomic hierarchal putative SCFA-producing genera *Faecalibacterium* (5 studies) (98, 108, 110, 113, 117), *Roseburia* (4 studies) (98, 103, 110, 111), *Blautia* (5 studies) (98, 103, 110, 113, 117), *Coprococcus* (2 studies) (98, 113), and *Dorea* (2 studies) (98, 113) (Supplementary Table 1).

Significantly, a few of the studies evaluated predicted functional gene content profiling (PICRUSt) (118) to infer changes in microbiota function. Keshavarzian et al. discovered PD subject's fecal samples had significantly higher abundant genes involved in lipopolysaccharide (LPS) biosynthesis, with a large number of genes involved in metabolism were significantly less abundant (98). Hill-Burns et al. indicated 17 upregulated pathways and 9 downregulated pathways, including xenobiotics degradation and metabolism of plant-derived compounds in PD subjects (110). Barichella et al. revealed 11 upregulated pathways and 15 downregulated pathways in *de novo* PD subjects, compared to healthy controls (111). Qian et al. predictive functional analysis indicated four metabolic pathways upregulated and 3 pathways downregulated (119). Finally, Bedarf et al. used the detailed metagenomics shotgun analysis to infer functional analyses of the metagenomes that showed differences in microbiota metabolism in PD subjects involving the β-glucuronate and tryptophan metabolism (109).

The intestinal microbiota does not appear to be the only microbiota that is disrupted in PD patients. To date, there are two studies that interrogated the nasal and oral microbiota community structure and composition in PD patients. Pereira et al. interrogated both nasal and oral microbiota profiles between PD patients and healthy controls (107). The oral microbiota composition was significantly altered in PD patients, compared to healthy controls, predominantly by higher relative abundance of opportunistic pathogens. The nasal microbiota lacked strong significant individual taxa differences, but trended toward an overall difference in the microbial composition between groups. In contrast, Mihaila et al. interrogated the oral microbiota using saliva samples through shotgun metatranscriptomic profiling and found significant changes in the microbiota community structure, composition and function in PD patients (106). The study found several similarities between dysbiotic oral microbiota and dysbiotic fecal microbiota in PD patients, when they compared their findings with previously published human PD studies. Dysbiotic oral microbiota once again was characterized by higher relative abundance of putative pro-inflammatory bacteria. This finding is potentially important in PD pathogenesis because one proposed site of initial injury in PD is the olfactory bulb, which is in close proximity to the oronasal space, as proposed by Braak et al. (120, 121).

However, the causal link between dysbiotic microbiota and the development of PD is yet to be established. The debate is whether these changes in microbiota community structure and composition in PD starts the trigger for PD, or are a consequence of PD. Indeed, several studies have shown a correlation between changes in microbiota and duration of the disease and dysbiosis is more pronounced in those with longer duration of PD (98, 104, 115, 117). This is not surprising because PD patients commonly change their life habit to better cope with their symptoms and this life style change can impact microbiota composition. For example, GI symptoms are common in PD patients (122, 123) and thus they typically change diet that could affect their microbiota. Although several studies did not find a correlation between diet and dysbiosis in PD patients numerous studies support a role for Western diet and possibly dairy products in PD risk (47, 51, 61, 124, 125). Constipation is very common in PD patients and typically occurs years before onset of CNS symptoms (123, 126, 127) and constipation can impact the microbiota community (128). However, dysbiosis was also found to occur in those PD patients who did not suffer from constipation (98). Patients with PD have poor sleep and reversal of sleep/wake cycles that can cause disruption of circadian rhythms (129, 130) and both disrupted sleep and circadian disruption can cause dysbiotic microbiota in both humans and rodents (131, 132). Additionally, PD medication correlates with dysbiosis (105, 110, 119). However, dysbiosis was still present in early onset and naïve PD patients on no PD medication (98). More importantly, dysbiosis has been reported in patients with idiopathic rapid eye movement sleep disorder (iRBD) (prodromal PD) (105). Thus, even though life style changes from PD symptoms and PD medication may contribute to changes in microbiota composition, it does not appear to explain the observed dysbiosis in PD patients. Taken together, these findings support the hypothesis that abnormal microbiota composition plays a critical role in the pathogenesis of PD and is a major contributor of symptomatic PD development.

One key question is how does the intestinal microbiota dysbiosis observed in multiple PD studies arise? The current model for a role for the microbiome in PD is that dysbiosis may be driving PD progression either via systemic inflammatory factors and/or increased α-Syn misfolding in the gut that results in aggregates of α-Syn being transported to the brain via the vagus nerve as hypothesized by Braak (95, 120). However, there is no established mechanism to explain the intestinal microbiome dysbiosis or even to what extent it is a consequence or cause of PD (95, 96, 133). Studies in which PD patient fecal transplant into genetic PD mice worsened the PD phenotype support a role for microbiome dysbiosis directly promoting PD progression (134). One possibility is a genetic contribution considering that LRRK2 polymorphisms are associated with PD risk and IBD risk and LRRK2 mediates microbial immune signaling. But the majority of sporadic PD appears to be associated with environmental risk factors that also affect the microbiome such as stress, diet, lack of exercise, and disruption of circadian rhythms seen in REM sleep behavior disorder (RBD) (4, 105, 135–137). Change in life style with PD that helps patients to cope with symptoms can affect microbiota-like lack of exercise and change in diet- these changes can explain worsening of dysbiosis in those with a long duration of PD but also in early onset PD (138). Gut dysfunction also can affect microbiota and constipation (128) could be a contributing factor and several studies link prodromal constipation with PD (123, 126). However, constipation cannot explain dysbiosis completely because PD patients without constipation still had dysbiosis and leaky gut (84, 98). Also, PD patients with RBD who had no constipation still exhibited dysbiosis (105). Thus, while the search goes on for mechanisms for PD dysbiosis, the most likely cause is Western lifestyle factors known to affect the microbiome including stress, Western diet, lack of exercise, and circadian disruption (4, 136).

## Microbiota-Gut-Brain Axis in PD

Recent models for PD pathogenesis have focused on the important role of the microbiota-gut-brain axis (MGBA). One school of thought, originally proposed by Braak et al. (120) actually proposes that PD originates in the GIT or possibly the nasal mucosa (121) and spreads to the brain (139, 140). In support of this model several studies have shown α-Syn protein exhibits prion-like properties and cell to cell transmission (141). Key papers showed inter-neuronal trans-synaptic transport of α-Syn in pathological studies in PD patients that had received striatal transplants supporting the spread of misfolded α-Syn to normal adjacent cells (142–144). Recent studies have now shown EE cells of the gut can produce misfolded α-Syn and synapse with enteric nerves to transmit α-Syn (14, 145). However, the true role of α-Syn in PD is still debated (146). The role of α-Syn in the intestine is discussed further below. The MGBA is the two-way communication between the GIT and the CNS/brain and consists of many mechanisms (6, 147, 148). The mechanisms of communication used by the MGBA include responses to bacterial components and bacterial metabolites (including pro-inflammatory products like LPS that could activate microglia and trigger neuro-inflammation) (149–151) and anti-inflammatory products like SCFA, especially butyrate (152), peptides [including neurotransmitters and neuromodulators such as g-aminobutyric acid (GABA)], serotonin, dopamine (151, 153) and hormones produced by cells of the GIT (154, 155). This interaction includes bidirectional microbiota–immune interaction and microbiota-nervous system interaction. In fact, a growing number of studies support two-way interaction of the microbiota with virtually every organ system (24). This bidirectional communication is increasingly acknowledged as playing an important role in brain function including in neurodegenerative diseases (147, 151).

Evidence supports that virtually every part of the GIT is affected in PD (122, 123). A pathologic hallmark of PD are so called Lewy bodies in the brain substantia nigra (SN) neurons that are found post-mortem. Lewy bodies are largely composed of the neuronal protein alpha synuclein (α-Syn). A key feature in PD is that aggregated and phosphorylated forms of α-Syn protein have also been observed in every major part of the GIT and enteric nervous system in patients with PD (84, 123, 156–158). For example, Lewy bodies/Lewy neurites are present in 72–100% of intestinal samples from PD subjects and 62% have phosphorylated α-Syn which is markedly greater than that observed in the healthy population (0–33% have α-Syn). These data suggest that intestinal synucleinopathy may be a relatively sensitive and reliable indicator of PD (123, 159). Importantly, increased phosphorylated α-Syn is also found in GIT tissues from prodromal PD patients suggesting that GIT involvement occurs early in disease pathogenesis (159, 160). This is supported by a recent study which reported that distinctive α-Syn immunoreactivity observed in intestinal biopsies collected from healthy individuals who would later go on to develop PD (156, 157). Taken together, these data support the idea that abnormal enteric α-Syn appears before neurodegeneration in CNS advances to a point that is sufficient for motor symptoms to emerge. Such data also support an intestinal origin for PD.

Motor impairments in PD are generally preceded by non-motor symptoms such as depression, olfactory deficits, sleep behavior disorder, and a number of GIT symptoms. The GIT symptoms can precede motor symptoms by more than 10 years and include GIT motility problems, colonic inflammation, and constipation (50–80%) (123, 127, 161). In fact, constipation is associated with a 2.7- to 4.5-fold increase in the risk of developing PD (123).

In 2003, Braak et al. postulated that an unknown pathogen (virus, bacterium) or toxin originating in the GIT or nasal passage/olfactory nerve (two hit hypothesis) could be responsible for the initiation of sporadic PD (120, 121). In this model of disease progression, the pathology initiates in the GIT (or nasal/olfactory) and propagates to the brain via the Vagus nerve or olfactory nerve (120, 162). Researchers have demonstrated that α-Syn fibrils, injected into the GIT mucosa of rodents, can propagate through the Vagus nerve and can be found in the brain (81, 163). Another recent study injected pre-formed α-Syn fibrils into the mouse stomach mucosa and found progressive PD pathology including α-Syn misfolding in the Vagus nerve and SN, an effect that was absent in vagotomized mice (80). With regard to vagotomy and risk of PD, a study by Svensson et al. found that full truncal vagotomy is associated with a decreased risk for subsequent PD, supporting that the vagal nerve may be critically involved in the pathogenesis of PD as proposed by Braak et al. (120, 164). However, two subsequent studies have disputed these findings. Tysnes et al. reanalyzed these data and found no significant risk reduction for PD with vagotomy (165). In addition, a second independent human study in Sweden found no decreased risk for PD after vagotomy (166). Thus, vagal involvement in PD disease development is still disputed (133).

However, even if the Vagus nerve isn't critical in initiating or promoting α-Syn PD pathology there are many other mechanisms by which the GIT can impact the brain via the vagus as we discuss below. The changes observed in the GIT in humans and animal models of PD are intriguing and begs further investigation into what is causing the GIT dysfunction and α-Syn aggregation to occur (140). One possible factor is the intestinal microbiota (95, 96).

A growing body of evidence now supports that the intestinal microbiota modulates behavior and contributes to neurological disorders and neurodegenerative diseases (151, 167–169). In fact, data show that the intestinal microbiota is necessary for the development of PD-like behavior and pathology in rodent models. Specifically, germ-free mice and antibiotic-treated mice have ameliorated PD-like behavior and pathology compared to their specific pathogen free counterparts (134). These data suggest that signaling between the microbiota and the brain is critical for PD-like outcomes in rodent models. It also appears that there is something remarkable about the PD microbiome that triggers events leading to neuroinflammation and neurodegeneration. Transfer of a microbiome from an MPTP-treated mouse into a control (non-MPTP) mouse is sufficient to induce motor impairment and activation of microglia and astrocytes in the SN (170). In addition, colonization of α-Syn-overexpressing (ASO) mice with microbiota from human PD patients enhances motor impairments compared to mice that received microbiota transplants from healthy human donors (134). These findings support that intestinal microbiome can regulate the development of PD-like pathology and behavior in mice and therefore may also be important in contributing to disease development in humans (95, 96). Perhaps PD should no longer be viewed solely as a complex disorder of motor functions, but rather as a progressive condition involving the GIT (6, 148, 171).

GIT-derived bacteria, bacterial components, and bacterial metabolites can trigger neurodegeneration through multiple pathways which are affected by diet and discussed below. First, is the intestinal barrier mechanism. In this mechanism, bacterial components (e.g., LPS) and bacterial metabolites (e.g., SCFA) produced by the microbiota influence intestinal barrier integrity which directly contributes to inflammation in the systemic circulation and in the brain (91, 137, 172). Second, is the NLRP3 inflammasome activation mechanism. Endotoxemia (i.e., LPS in the blood) resulting from barrier dysfunction activates the NLRP3 inflammasome and results in mitochondrial dysfunction and IL-1b production and insulin resistance with important consequences for neuronal function (77). Finally, are the intestinal peptide and intestinal gluconeogenesis mechanisms (173, 174). Bacterial metabolites influence the production of the GIT peptide production, insulin resistance, mitochondrial function, and vagal stimulation of brain derived neurotrophic factor (BDNF) production in the brain. This list of potential mechanisms is by no way means exhaustive but reflects key topics that are rapidly emerging as factors contributing to diet-microbiome regulation of gut-derived inflammation in neurodegeneration and PD.

## Intestinal Barrier Mechanism

The intestinal epithelial barrier separates the pro-inflammatory luminal contents (e.g., LPS) from reaching the intestinal and systemic circulation, and the intestinal microbiota is a critical regulator of intestinal barrier integrity (91, 175). Intestinal barrier dysfunction (i.e., intestinal leakiness) has been observed in newly diagnosed, untreated PD patients which is also associated with increased LPS staining and α-Syn aggregates in the colonic mucosa (84). GIT dysfunction has also been described in animal models of PD including in both genetic and toxin-induced models (122, 123) which occurs concurrently with α-Syn aggregations in the GIT (123). These observations further support the hypothesis that PD may originate in the GIT (139).

Indeed, intestinal microbiota dysbiosis (especially when characterized by a reduction in SCFA-producing bacteria that has been reported in PD patients) is associated with intestinal barrier dysfunction and endotoxemia (i.e., LPS in the blood) (91, 95). Specifically, bacterial production of SCFA appear to be critically important in regulating the barrier (87). The three principal colonic SCFA include acetate (2-carbon), propionate (3-carbon), and butyrate (4-carbon). These typically exist in the colon in a millimolar ratio of 60:20:20 (acetate:propionate:butyrate) (176). Two other important SCFA receiving are lactate and succinate. SCFA exert beneficial effects through multiple mechanisms (87). Previous reviews of SCFA mechanisms have focused on SCFA specific GPCR signaling via specific receptors: GPR41 (propionate/butyrate), GPR43 (acetate/propionate), and GPR109a (butyrate) for acetate, propionate, and butyrate (87, 177). Also GPR81 (lactate) and GPR91 (succinate) have received recent attention (87). These GPCR for SCFA are reviewed in detail elsewhere (87, 177). Broadly speaking, SCFA GPCR positively modulate immunity and anti-inflammatory signaling in immune and other cells as well as mitochondrial cellular metabolism (178, 179). Butyrate (and to a lesser extent propionate and acetate) also has histone deacetylase inhibitor (HDACi) activity that can have epigenetic effects on gene expression, and butyrate is used by colonocytes as an energy source (10, 177). It is through these mechanisms that SCFA (especially butyrate) influences intestinal barrier integrity. Indeed, a reduction in putative SCFA-producing bacteria or a reduction in luminal SCFA (due to intestinal microbiota dysbiosis) is associated with intestinal barrier dysfunction (10, 87, 180).

Diet-induced dysbiosis or even age-associated dysbiosis (a normal feature associated with aging) (91, 175), are characterized by a loss of SCFA-producing bacteria and SCFA, these may be able to trigger intestinal barrier dysfunction and subsequent inflammatory events leading to systemic inflammation as well as neuroinflammation and neurodegeneration (101, 175). Newly diagnosed, treatment naive PD subjects have evidence of intestinal barrier dysfunction compared to age matched controls (84, 181). Specifically, PD subjects have elevated levels of serum LPS binding protein (LBP, binds to LPS to elicit an immune response), abnormal intestinal tight junction proteins, fecal markers of leaky gut, serum zonulin, as well as *E. coli* in the intestinal mucosa compared to age matched controls (84, 181, 182). In support of intestinal barrier dysfunction being a critical mechanism, diseases characterized by intestinal microbiota dysbiosis and barrier dysfunction are a risk factor for developing PD. Specifically, Four studies in patients with inflammatory bowel disease (IBD), which is also characterized by intestinal microbiota dysbiosis and barrier dysfunction, support a significantly increased risk for developing PD compared to people without IBD (183–186). Also, a recent systematic review and meta-analysis of these four IBD-PD studies above concluded that the overall risk of PD in IBD was significantly higher than controls. Crohn's disease had a 28% increased risk of PD and ulcerative colitis had a 30% increased risk of PD compared to controls (187). In support of these data two studies using the DSS rodent model of ulcerative colitis concluded that DSS in drinking water and the resulting intestinal inflammation exacerbated symptoms of PD in both the LPS-striatum injection PD model (188) and an α-Syn overexpressing genetic PD model (189). However, in one recent US study using a large Medicare database analysis and newly diagnosed PD patients, IBD was associated with lower risk of PD as were Crohn's disease and Ulcerative colitis individually (190). The reasons for these differences are not clear and the role of IBD in PD risk remains to be defined. Studies have also demonstrated that a genetic variant that is a risk factor for IBD (leucine rich repeat kinase 2, LRRK2, important in the response to microbial ligands), is also a risk factor for PD (191). Furthermore, restraint stress (which caused intestinal barrier leak) exacerbated PD-like symptoms and loss of dopaminergic neurons in the striatum in the rotenone rodent model of PD (137).

Endotoxin in the blood (as a consequence of intestinal barrier dysfunction) can affect the brain directly (101, 149, 175). Like PD, Alzheimer's disease (AD) is a neurodegenerative disease that is also characterized by intestinal microbiota dysbiosis and barrier dysfunction (192, 193). Recent post mortem analysis of AD patient brains reveals LPS staining in the hippocampus and cortex of AD patients is 21-fold greater than that observed in control brain tissue (150, 194). Like AD, PD is also characterized by intestinal barrier dysfunction and endotoxemia (84), therefore it is possible that intestinal barrier dysfunction may play a key role in PD development and/or progression (95).

Mechanistically, Western diet dysbiosis, intestinal barrier dysfunction and endotoxemia can lead to immune activation and neuroinflammation (91, 101, 195–197). Toll like receptors (TLRs) recognize pathogen associated molecular patterns (PAMPs) located on the surface of bacteria (198). Among the most widely studied is the interaction between TLR4 and LPS (199). TLRs are located on a wide variety of cell types and are critical to mount an appropriate immune response to bacteria. In fact, administration of systemic LPS has been used as a model for PD for many years (149, 197, 200). Mechanistically, this appears to be the consequence of LPS-driven activation of TLR4, especially on brain microglia (201). Specifically, TLR4 knock out mice are protected from the effects of oral low dose rotenone as well as MPTP including less neuroinflammation and neurodegeneration, compared to rotenone-treated, wild-type mice (68, 202). These data support that TLR4 receptors are important in the development of PD-like pathology.

Taken together, it appears that barrier dysfunction, leading to endotoxemia, and TLR4 receptor activation may result in a series of events culminating in systemic inflammation and neuroinflammation and neurodegeneration (91, 101, 203–205). Even if intestinal barrier dysfunction is a consequence of PD (and not an initiating trigger/cause), intestinal barrier dysfunction and the resulting endotoxemia may still produce sustained neuroinflammation that promotes PD disease progression (101, 203).

## NLRP3 Inflammasome Activation Mechanism

One of the consequences of TLR activation is microglial NLRP3 inflammasome activation (77, 206). In response to activation of TLRs, the NLRP3 inflammasome assembles and produces inflammatory cytokines (207, 208). Among the most widely studied inflammasomes is the NLRP3 inflammasome which produces pro-inflammatory cytokines especially IL-β as well as IL-1α, IL-18, and IL-33 (209). Inflammasomes are present in peripheral immune cells such as macrophages, as well as in the brain and especially in microglia (206, 210, 211). A role for microglial NLRP3 inflammasome in PD has recently been proposed (77). The NLRP3 inflammasome has also emerged as a potential driver of α-Syn neuroinflammation in PD (212). The current model of NLRP3 activation proposes a “two signal” model (213). In this model, TLR signaling is the first signal which induces NF-kB-mediated expression of pro-IL-1β and pro-IL-18. The second signal can be ATP, calcium or potassium flux or mitochondrial reactive oxygen species (**ROS**) which can occur as a consequence of a number of factors such as intestinal microbiota dysbiosis, endotoxemia (11, 213–217) or other factors that induce mitochondrial dysfunction such as aging (60, 218). Another possible second signal is misfolded α-Syn (aggregated α-Syn) that was induced by TLR/NF-kB mediated inflammation (77, 212). The second signal induces NLRP3 assembly and subsequent caspase-1 activation. The combination of the first and the second signals results in cleavage of pro-IL-1β to its active form IL-1β (and other cytokines like IL-18) (213) which has a wide range of biological consequences including creation of sustained pro-inflammatory/oxidative stress in the brain that would lead to more α-Syn aggregation, more neuro-inflammation enough to cause DA loss and neurodegeneration and symptoms of PD (77).

There is a substantial amount of data demonstrating the importance of the NLRP3 inflammasome in PD. Recent post mortem studies in PD patients show that the NLRP3 inflammasome is significantly upregulated in the SN of PD patients (almost entirely localized to microglia) (77). This upregulation in NLRP3 was also observed in mouse models of PD and AD (77, 219) and it appears to be important in disease pathogenesis. Specifically, inhibition of NLRP3 protects against neurodegeneration in all rodent models of PD tested including injection of pre-formed α-Syn fibrils (PFF), rotenone, and MPTP models (77, 215, 220). Similarly, knocking out NLRP3 in an AD animal model (another neurodegenerative disease) protects mice from developing AD-like behavior and brain pathology (219). Thus, activated NLRP3 inflammasome appears to be a key driver of neuroinflammation in PD (77, 220). In addition, NLRP3 levels also appear to increase with other factors such as age and consumption of a Western diet, it could be that the increase in NLRP3/IL-1b reduces the resiliency of the brain to respond to a secondary insult such as gut-derived endotoxemia from microbiota dysbiosis and/or intestinal barrier dysfunction (11, 221, 222).

In addition to LPS activation of TLR4, the microbiota can also influence the NLRP3 inflammasome by producing secondary bile acids. Primary bile acids are produced in the liver and are subsequently released into the GIT to aid in the digestion and absorption of lipids. Most primary bile acids are absorbed in the small intestine but those that reach the colon are metabolized by the intestinal microbiota to form secondary bile acids. Importantly, secondary bile acids can inhibit the NLRP3 inflammasome via the TGR5 receptor and are dysregulated in Western diet induced dysbiosis (223, 224).

As already mentioned, NLRP3 activation results in production of several cytokines but perhaps the one that may be most relevant for PD is IL-1β. IL-1β is not only a potent pro-inflammatory cytokine and thus a major player in neuro-inflammation in PD, but also has many other biological effects. Among the many consequences of IL-1β production is the development of insulin resistance (218). Specifically, IL-1β blocks signaling associated with insulin receptors. Activation of NLRP3 and subsequent IL-1β production are the single greatest factors that drive insulin resistance, and NLRP3 KO mice are protected from developing insulin resistance (225, 226). Specifically, cytokines, especially IL-1β, block insulin signaling which has important detrimental consequences on neuronal mitochondrial function and cellular health. In fact, insulin resistance is characteristic of both the PD and AD brain (227–229) and diabetes is a risk factor for development of PD (228).

Insulin resistance and type 2 diabetes mellitus (T2DM, characterized by insulin resistance) may cause neuroinflammation by driving mitochondrial dysfunction, leading to excessive production of ROS, cellular stress, NLRP3 activation and neuroinflammation (especially via microglia), ultimately culminating in neuronal dysfunction and death (228, 229). Insulin resistance is commonly observed during aging, but it may also be important in the pathogenesis of PD (229). The incidence of both T2DM and PD are both increasing in Western societies suggesting that these two diseases may be related (230). In fact, as noted, T2DM is a risk factor for PD and is characterized by intestinal microbiota dysbiosis similar to that observed in PD (loss of SCFA-producing bacteria, increase in LPS-containing bacteria) (231–236). Premature cognitive decline is also a feature commonly observed in patients with T2DM (231). Inhibition of NLRP3 (via glyburide or pioglitazone, the SCFA butyrate, or MCC950) prevents the development of insulin resistance and T2DM as well as PD (77, 211, 237–240). Taken together, these data support a model for a cascade of events culminating in intestine-derived neuroinflammation and neurodegeneration. Specifically, LPS-TLR activation of the NLRP3 inflammasome induces production of IL-1β resulting in insulin resistance, mitochondrial dysfunction, and ROS production, further NLRP3 activation and neuroinflammation and neurodegeneration.

## Intestinal Peptide and Intestinal Gluconeogenesis Mechanisms

Influence of diet and the intestine on brain function (gut-brain axis) is not necessarily limited through intestinal microbiota. The intestine produces a number of substances that directly or indirectly influence the brain. These substances are produced in response to dietary components (e.g., fats) but also are produced in response to bacterial metabolites. Bacterial products, SCFA and secondary bile acids, can both promote the production of the incretin hormones glucagon-like peptide-1 (GLP-1) and glucose dependent insulinotropic polypeptide (GIP) by L-cells of the GIT (87, 241–243). GLP-1 and GIP impact a number of cell types that can directly or indirectly affect neuroinflammation and neurodegeneration in PD.

GLP-1 has multiple mechanisms of action. One important consequence of GLP-1 production is reduced inflammation. For example, stimulation of the GLP-1 receptor (via GLP-1 or agonists) inhibits the NLRP3 inflammasome (244–246). In so doing, GLP-1 prevents the cascade of events including IL-1β production culminating in insulin resistance, mitochondrial dysfunction and cellular stress. GLP-1 also corrects insulin resistance by stimulating pancreatic cells to produce insulin and normalizing insulin signaling and mitochondrial function in brain neurons (247). Normalizing insulin resistance improves mitochondrial function and reduces ROS production, which has the net effect of blocking neuroinflammation and improving neuronal health. GLP-1 can have effects within the brain itself because it can cross the blood brain barrier and receptors for GLP-1 are located on neurons, astrocytes, and microglia (247–249). GLP-1R-deficient mice show impaired performance in memory-related behavioral tasks (248). In addition, GLP-1 is protective against neuronal apoptosis in the Alzheimer's disease model (247). Finally, stimulation of GLP-1 receptors induce production of BDNF in the brain and also stimulate vagal signaling from the gut to further promote brain BDNF (247, 248). BDNF is a critical factor for survival and health of dopaminergic neurons in the SN (250). Indeed BDNF is dramatically decreased in PD brain tissue, thus, the ability to increase BDNF is an important consequence of GLP-1 production (250, 251).

Alterations in GLP-1 signaling are associated with many features associated with PD or risk factors for developing PD. For example, intestinal microbiota dysbiosis disrupts normal GLP-1 signaling (252), reduced GLP-1 production is associated with metabolic syndrome (insulin resistance) (253), and reduced GLP-1 is associated with reduced BDNF in the brain (254). On the flip side, GLP-1 agonists are protective in several rodent models of PD (174, 247). Agonists of GLP-1 and dual treatment of GLP-1/GIP demonstrate neuroprotection in MPTP models of PD (255, 256). It is possible that these effects are mediated through a mechanism involving both inhibition of NLRP3 and an increase in the production of glial derived neurotrophic factor (GDNF) and BDNF and may involve GLP-1 induced improvement in insulin sensitivity as well as GLP-1 vagal stimulation (174). Importantly, recent clinical trials show that GLP-1 agonists elicit significant improvements in PD patient disease scores compared to placebo (248, 257, 258).

Intestinal gluconeogenesis (IGN) is also a mechanism by which the diet and microbiota can influence neuroinflammation and neurodegeneration. Recent studies have shown that the SCFA (butyrate, propionate) can regulate host metabolism by stimulating IGN in intestinal epithelial cells that in turn promotes vagal signaling (173). It should not be surprising then that a healthy high fiber diet and increased gut SCFA can correct insulin resistance via both IGN-vagal-BDNF signaling and by GLP-1/GIP stimulation and preventing intestinal leakiness and NLRP3 activation (10, 87, 259). IGN vagal BDNF stimulation is a key mechanism by which IGN may promote normal brain glucose metabolism which is dysregulated in PD (173). Thus, IGN from gut SCFA can also influence BDNF production in the brain via the vagus (260). BDNF promotes neuronal cell health and normal insulin signaling in the brain (261). It makes sense then that impaired insulin sensitivity in the PD brain is associated with low BDNF levels (250, 262–264).

There are multiple mechanisms by which GLP-1, GIP, and IGN can influence the brain but it is interesting that they all share the feature of being able to upregulate production of BDNF (262). BDNF is also a key neurotrophic factor in CNS degeneration and regeneration (262). Reduced levels of serum BDNF are observed in PD patients compared to healthy controls, including in the serum and in the brain (SN, caudate-putamen) (251, 265, 266). It is intriguing to think that Western diet intestinal microbiota dysbiosis leading to low SCFA production might blunt the expression of BDNF through a mechanism involving gut leakiness and loss of GLP-1, GIP, and/or IGN. Western diet dysbiosis also results in loss of (fewer) gut vagal afferents in rats (267). Finally, it is noteworthy that GLP-1, GIP, and IGN and other intestinal hormones are largely influenced by diet and dietary intervention such as switching from primarily animal-based Western diet to primarily plant-based diet can promote normal homeostasis of these hormones. These data are yet another scientific rationale for considering dietary intervention to prevent/treat or at least modify disease course in PD.

## Diet as a Prevention or Treatment for PD

Based on these data it is clear that there are several mechanisms by which intestinal bacteria, bacterial products, or bacterial metabolites and intestinal hormones can influence neuroinflammation and neurodegenerative processes. Therefore, it seems logical that dietary interventions targeted at modifying the intestinal microbiota structure and/or function and intestinal peptides may modify PD disease pathogenesis. Indeed, Hippocrates' said: “Let food be thy medicine and medicine be thy food” (10). Diet has recently gained importance as a risk factor for developing PD and also as a potential therapeutic approach to treat PD (6, 7, 268). Below is a summary of dietary interventions that may be useful in the prevention and/or treatment of PD as well as the mechanisms by which this benefit may be conferred on the brain.

## Mediterranean Diet as a Treatment

The main components of the Mediterranean diet (MedD) include: daily consumption of vegetables, fruits, nuts, whole grains, and healthy fats; weekly consumption of fish, poultry, beans, and eggs; moderate consumption of dairy products; and limited intake of red meat (10, 124). Adherence to the MedD is associated with decreased risk of PD (9, 269, 270). One of the most dramatic differences between the traditional Western diet and the MedD is dietary fiber intake. Consumption of dietary fiber is typically very low (<10–15 g/day) in Western societies, but high (>25–30 g/day) in those who consume a Mediterranean diet (10, 87–89). It makes sense then that the Mediterranean diet-associated microbiome is characterized by a high relative abundance of bacteria that can utilize fiber as an energy source such as SCFA-producing bacteria (10, 89). Indeed, microbiota communities from subjects consuming a Mediterranean diet are enriched in SCFA-producing bacteria (10, 87, 89, 271). Fiber can also be administered experimentally to alter the microbiota structure and function including an increase in the relative abundance of fiber-fermenting (“good”) bacteria as well as increased production of SCFA (10, 87).

These microbiome changes can elicit a myriad of effects that are beneficial in blunting neuroinflammation and PD pathogenesis. For example, consumption of a high fiber diet improves intestinal barrier function and insulin resistance, improves insulin sensitivity, increases GLP-1/GIP production, stimulates IGN, and increases brain BDNF production (173, 259, 272, 273). Conversely, when fiber consumption is low, the microbiota instead use protein as an energy source which favors the growth of gram negative (LPS-producing, dysbiosis) bacteria and the production of metabolites such as branched chain fatty acids including isovalerate and 2-methyl butyrate that have been associated with insulin resistance (a feature of PD) (274). Fiber consumption (and the consequent production of SCFA) is one mechanism by which the Mediterranean diet may beneficially impact PD development and progression.

In addition to fiber, the Mediterranean diet is also rich in foods that contain anti-oxidant bioflavonoids and polyphenols, which are associated with decreased risk of PD (9, 35, 270). Flavonoids are typically found in fruits, vegetables, grains, and tea. There are not a lot of data available, but it appears that flavonoid consumption also may trigger an increase in SCFA production (36) and several polyphenol bioflavonoids (including in coffee) and fish oil are associated with inhibition of the NLRP3 inflammasome (33, 275). Also, nuts and olive oil stimulate GLP-1 secretion and the MedD after 28 days has been shown to increase GLP-1 production (241).

Taken together there are multiple mechanisms by which the Mediterranean diet can beneficially impact the brain. There is a common theme that components of the Mediterranean diet are especially able to alter the microbiota in a way that promotes SCFA production. SCFA can influence so many PD relevant mechanisms such as barrier function, mitochondrial function, NLRP3, and intestinal peptide production (259, 272, 273) and vagal stimulation of BDNF and thus might be beneficial in PD. However, to date there is no high-quality clinical trial to test the potential benefit of a high fiber Mediterranean diet in PD. These data above provide a strong scientific rationale for conducting randomized controlled dietary trials in PD to determine whether Mediterranean diet can impact neuroinflammation and disease course of PD patients.

## Ketogenic Diet and Fasting as a Treatment

It is well-established that caloric restriction and/or intermittent fasting are anti-inflammatory processes and can ameliorate disease in a variety of experimental models, including PD (276, 277). Intermittent fasting is a feeding regimen that cycles between periods of fasting (with either no food or significant caloric restriction), and periods of unrestricted eating. Caloric restriction can improve health, increase lifespan, and improve tolerance to metabolic stresses (278, 279). Indeed, rodents on an intermittent fasting diet exhibit less neuronal dysfunction/degeneration, and fewer PD-like symptoms in models of PD compared to *ad libitum*-fed controls (280). Similarly, caloric restriction increases levels of neurotrophic factors such as BDNF and attenuates PD-like pathology (including dopaminergic neuron loss) and behavior in rodent and primate models of PD (281, 282) lifestyle interventions such as caloric restriction/fasting and ketogenic diets are currently used to treat epilepsy and other neurological diseases (278, 279). These effects may be due to the fact that ketosis (due to caloric restriction/intermittent fasting, ketogenic diet) increase neurotrophic factors such as BDNF, increases levels of antioxidants, and reduces pro-inflammatory cytokine production (280, 282).

Both fasting and consumption of a ketogenic diet (55–60% fat, 30–35% protein, 5–10% carbohydrate) result in the production of ketone bodies (283). Two metabolic processes are critical in producing energy: gluconeogenesis and ketogenesis. Gluconeogenesis is the endogenous production of glucose in the body primarily from lactic acid, glycerol, and the amino acids alanine and glutamine. When glucose levels are low for prolonged periods (as with fasting), the endogenous production of glucose is not able to keep up with the needs of the body and ketogenesis is primarily used to derive energy (8, 278). Fatty acids and some amino acids are metabolized to form basic ketone bodies which accumulate in the body including: acetoacetate, beta-hydroxybutyrate (BHB), and acetone (8, 278). Ketone bodies may play an important role in mediating the beneficial effects of intermittent fasting and the ketogenic diet on the brain (276).

Ketone bodies are beneficial in humans with PD and animal models of PD. One early study found beneficial effects of hyperketonemia on PD patients (284). Likewise, in a rodent model of PD, BHB is associated with protection against MPTP-induced damage to dopaminergic neurons (285). Furthermore, BHB injection into the brain can rescue mitochondrial function and ameliorate dopaminergic neurodegeneration and motor deficits induced by MPTP in mice (286).

The effects of ketone bodies may be the consequence of a wide variety of mechanisms. For example, ketone bodies can cross the blood brain barrier and may bypass the type 1 complex mitochondrial defect in PD to rescue mitochondrial ATP function (8, 278). Another intriguing potential mechanism is the effects of ketone bodies on the NLRP3 inflammasome (287). For example, fasting can inhibit NLRP3 activation, which is thought to be due to effects of BHB (288, 289). Indeed, BHB directly inhibits the NLRP3 inflammasome and attenuates NLRP3-mediated inflammatory disease (287, 290). Likewise, fasting MPTP mice decreases IL-1β, a marker for NLRP3 activation (218).

In addition to ketone bodies, fasting and consumption of a ketogenic diet can also impact PD pathogenesis by influencing intestinal peptide production (i.e., GLP-1 and GIP) with downstream effects on NLRP3 inflammasome, insulin resistance, and BDNF production (276). Indeed, caloric restriction increases brain BDNF in a primate model of PD (282). Recent studies in MPTP mice shows that fasting increases BDNF in the brain (276).

Also, it appears that fasting impacts normal insulin signaling. Every other day fasting also corrects insulin resistance/T2DM in mice (291). This affect appears to be specific to changes in the intestinal microbiome, including the production of SCFA. Transfer of stool from mice fed every other day into mice with T2DM was sufficient to improve insulin resistance in the recipient mice similar to that observed due to every other day fasting itself (291). Thus, microbiota SCFA, IGN, and/or GLP-1 mediated mechanisms discussed above may play a role in the fasting effects as well. Intermittent fasting also promotes secondary bile acid production and improves intestinal barrier function in mice by restructuring the microbiome to produce more SCFA (292). Finally, ghrelin is another intestinal peptide that is produced in response to fasting and ghrelin is neuroprotective in the PD MPTP model (293). It is thought that the ghrelin protective mechanism may be by promoting mitochondrial health and preventing NLRP3 IL-1β production (293–295).

Collectively, there is evidence that fasting and a ketogenic diet might be beneficial in PD and this effect may be mediated in significant part by changes in the intestinal microbiota. However, once again a well-designed trial is needed to show if the ketogenic diet is beneficial in PD before any serious consideration of fasting/ketogenic diet in the clinical care of PD patients.

## Omega 3 Polyunsaturated Fatty Acids

Consumption of PUFAs is also an element of the Mediterranean diet and generally protective against neurodegeneration in AD or PD (296). There are three principal types of omega-3 (ω3) PUFAs including eicosapentaenoic acid (EPA), docosahexaenoic acid (DHA, typically from fish oil), and alpha-linolenic acid (ALA) (296). Dietary supplementation with PUFAs reduces depression in PD patients, which is important because depressive symptoms are common in PD patients and often impact other clinical aspects of the disease (297). In addition, EPA are neuroprotective in several neurodegenerative diseases including PD (6, 18, 298–300). Rodent models of PD also show benefit of PUFA administration. Consumption of an EPA-enriched diet lessens MPTP-induced movement dysfunction (i.e., hypokinesia) and ameliorates memory deficits in mice (298, 301). Administration of DHA reduces 6-OHDA-induced behavior deficits (i.e., ipsilateral rotations) and increases tyrosine hydroxylase (the enzyme required to produce dopamine) levels in a PD rat model (302). Experimentally, DHA is often combined with uridine monophosphate (UMP, a dietary precursor for membrane phospholipid synthesis), the DHA/UMP combination prevents the development of PD-like behavior and pathology in oral and striatal administration of rotenone models (61, 303). In addition, DHA/UMP combination reduces parkinsonian-like behaviors and elevates dopamine levels in 6-OHDA treated rodents (302). There are many mechanisms by which ω3 fatty acids may impact the brain and be beneficial in the prevention and/or treatment of PD. *GLP-1 stimulation*: As noted above, fish oil and olive oil can stimulate GLP-1/GIP production by the intestine (241). *Cell Death*: Studies have revealed that supplementation with EPA or DHA attenuates dopaminergic cell death induced by MPTP administration (301, 304). DHA may protect neurons against cytotoxicity through a variety of mechanisms such as inhibition of nitric oxide production, inhibition of caspase signaling pathways (305), inhibition of tau hyperphosphorylation (306), as well as regulation of other signaling pathways (e.g., PI3K/Akt). *Cell Function*: In addition to inhibiting neuronal cell death, DHA promotes optimal dopaminergic structure and function including synaptic plasticity (synapse formation, dendritic spine density) and dopaminergic neurotransmission (303, 307). *Inflammation*: The protective effects of DHA may be mediated by a metabolic derivative known as neuroprotectin D1 (NPD1) (308, 309) which is an inhibitor of NLRP3 (310). Indeed, NPD1 protects neurons against oxidative stress, inflammation, and from activation of apoptotic signaling pathways. Thus, while Western diet saturated fats activate the NLRP3 inflammasome (11), consumption of ω3 fatty acids inhibit the NLRP3 inflammasome (including in brain microglia) probably via a mechanism involving reduced mitochondrial stress (311–313). It should not be surprising then that ω3 fatty acids prevent NLRP3 inflammasome-dependent inflammation and insulin resistance in a T2DM rodent model (314). *Other*: DHA may also protect the brain by increasing glutathione reductase activity essentially preventing protein oxidation (315, 316), lipid peroxidation, and the production of ROS (317). Other potential mechanisms of action of DHA include regulation of NF-κB activation, transcription modulation, and cell membrane properties (318, 319). Again, these data provide a strong scientific rationale for conducting randomized controlled dietary trials in PD to determine whether PUFA supplements can impact neuroinflammation and the disease course of PD patients before recommending it to PD patients.

## Conclusion

There is a growing body of experimental *in vitro, in vivo* animal and epidemiological evidence strongly suggesting that diet impacts the development/progression of multiple neurodegenerative diseases including PD. This includes both beneficial effects of diets rich in fiber, bioflavonoids, and ω3 fatty acids (e.g., the Mediterranean diet), and fasting and the ketogenic diet due the production of ketone bodies as well as the collective detrimental effects of the Western diet that include gut leakiness, NLRP3 activation, insulin resistance, and lack of beneficial SCFA/GLP-1 vagal signaling due to low fiber content. As we have discussed many of these effects may be due in large part to beneficial or negative effects on the intestinal microbiota. Diet rapidly and robustly alters the intestinal microbiome; thus, it is possible that these effects of diet are mediated (at least in part) by changes in microbiota structure and or function.

We described a mechanism by which intestinal dysbiosis can trigger intestinal barrier dysfunction leading to gut-derived LPS with systemic and neuroinflammation. We also described how bacterial components such as LPS can serve as a first signal in NLRP3 inflammasome mediated production of IL-1β, insulin resistance, and mitochondrial dysfunction. Finally, we described how bacterial metabolites such as SCFA and secondary bile acids can directly improve mitochondrial health as well as influence the production of the intestinal peptides GLP-1 and GIP that can directly promote brain health and stimulate IGN and together also regulate vagal stimulation of BDNF in the brain as well.

These data suggest that consumption of a Mediterranean diet might be a useful approach to prevent and possibly treat PD. This is because the characteristic features of the Mediterranean diet including high dietary fiber, bioflavonoids, and ω3 fatty acids that will modulate the microbiome and intestinal cell signaling and result in several alterations that confer benefits in the brain such as improved intestinal and blood brain barrier function, decreased NLRP3 inflammasome activation and IL-1β production, improved insulin sensitivity, increased GLP-1/GIP, IGN vagal stimulation, and increased production of BDNF in the brain. Even if not adhering to the Mediterranean diet, including dietary supplements for dietary fiber, bioflavonoids, or ω3 fatty acids may be beneficial. Similar benefits may be obtained by following a diet involving intermittent fasting or a ketogenic diet.

Further investigations into the mechanisms by which the intestinal microbiota contributes to the development and progression of PD are warranted. More importantly, there is a major unmet need to determine whether dietary intervention can prevent progression of PD from the prodromal phase to the overt CNS/motor phase and whether dietary intervention can modify disease course and disease progression (and response to levodopa treatment) in those who suffer from motor symptoms. We believe that the experimental data and epidemiological findings discussed above provided a strong scientific rationale to conduct well-designed dietary and intestinal microbiota-directed randomized control trials (RCT) in both prodromal and established PD patents.

## Author Contributions

All authors contributed significantly to the writing of this review. AJ, CF, AK, RV, PE, and VR wrote the first draft. MS edited the first draft and the final version. All authors edited and approved the final draft. All authors edited and contributed to the final revised resubmission.

### Conflict of Interest

The authors declare that the research was conducted in the absence of any commercial or financial relationships that could be construed as a potential conflict of interest.
