# Supplementary material for: Diet in Parkinson's Disease: Critical Role for the Microbiome
Source: Front Neurol. 2019 Dec 10;10:1245. doi: 10.3389/fneur.2019.01245 (PMC6915094; doi:10.3389/fneur.2019.01245)
Supplement: Supplementary file 1 [file Data_Sheet_1.PDF]

Table 1. Microbiota Alterations in Human Parkinson's Disease Clinical Studies.				
**Investigators**	**Discovery**	**Study Design**	**Sequencing Methodology**	**Significant Individual Taxa Abundance Findings (↑:increase) or (↓:decrease)**
Scheperjans et al. 2015	In comparison to healthy controls, patients with PD had significantly altered GI microbial compositions, specifically demonstrating a reduction in Prevotellaceae family, thus indicating increased gut permeability.<br><br>PD patients with severe postural instability and gait difficulty displayed a higher abundance of Enterobacteriaceae family.	Case-Control study<br><br>PD (N=72)<br><br>Random Healthy Controls (N=72)	16S rRNA V1-V3 gene amplicon sequencing<br><br>Fecal samples	**Proteobacteria phylum:**<br>↑Bradyrhizobiaceae family<br>**Firmicutes phylum:**<br>↑Lactobaillaceae family,<br>↑Clostridiales Incertae Sedis IV family<br>**Verrucomicrobia phylum:**<br>↑Verrucomicrobiaceae family<br>**Bacteroidetes phylum:**<br>↓Prevotellaceae family
Keshavarzian et al. 2015	PD patients demonstrated significantly altered GI microbial composition in fecal and mucosal communities in comparison to healthy controls.<br><br>PD duration had positive correlations with Bacteroidetes and Proteobacterium phylum and negative correlations with Firmicutes phylum, Lachnospiraceae family, and Blautia genus. This further suggests the presence of pro-inflammatory dysbiosis in PD patients, potentially triggering the misfolding of α-synuclein protein.<br><br>Predictive functional analysis demonstrated a higher abundance of pathways including LPS biosynthesis, ubiquinone and other terpenoid-quinone biosynthesis, and type III secretion systems within fecal samples of PD patients.	Case-Control study<br><br>PD (N=38): Untreated naïve (N=12), Treated (N=26)<br><br>Random Healthy Controls (N=34)	16S rRNA V4 gene amplicon sequencing<br><br>Fecal and Colonic Sigmoid Mucosa samples	**Mucosa:**<br><br>**Firmicutes phylum:**<br>↓Faecalibacterium genus,<br>↓Dorea genus<br>**Proteobacteria phylum:**<br>↑Oxalobacteraceae family,<br>↑Ralstonia genus<br>**Actinobacteria phylum:**<br>↓Coprobacteriaceae family<br><br>**Feces:**<br><br>**Ratio of Firmicutes-to-Bacteroidetes was significantly higher in PD patients.**<br><br>**↑Bacteroidetes phylum:**<br>↑Bacteroidaceae family,<br>↑Bacteroides genus<br>**↑Proteobacteria phylum**<br>**↑Verrucomicrobia phylum:**<br>↑Verrucomicrobiaceae family,<br>↑Akkermansia genus<br>**Firmicutes phylum:**<br>↑Clostridiaceae family,<br>↓Lachnospiraceae family,<br>↓Coprobacillaceae family,<br>↑Oscillospira genus, ↓Blautia genus,<br>↓Coprococcus genus,<br>↓ Roseburia genus, ↓Dorea genus
Hasegawa et al. 2015	In comparison to the control cohort, patients with PD exhibited an altered gut microbial composition, specifically an increase in abundance of Lactobacillus genus.<br><br>Disease duration had a positive correlation with Lactobacillus gasseri species and a negative correlation with Clostridium coccoides.	Case-Control study<br><br>PD (N=52)<br><br>Random Healthy Controls (N=36)	Real- time Quantitative PCR 16s rRNA or 23S rRNA<br><br>Fecal samples	**Firmicutes phylum:**<br>↑Lactobacillus genus,<br>↓Clostridium coccoides species,<br>↓Clostridium leptum species<br>**Bacteroidetes phylum:**<br>↓Bacteroides fragilis species

Unger et al.<br>2016	PD patients exhibited significantly altered GI microbial compositions when compared to healthy controls.<br><br>Metabolomics of SCFA in PD patients demonstrated a reduction in absolute concentrations of butyrate, acetate and propionate, and a reduction in the relative concentrations of butyrate.<br><br>Metabolomics of SCFA of PD patients taking COMT inhibitor medication demonstrated a reduction in absolute and relative concentrations of butyrate.	Case-Control study<br><br>PD (N=34)<br><br>Random Healthy Controls (N=34)	Real time-Quantitative PCR<br><br>Fecal samples	**PD:**<br><br>**Firmicutes phylum:**<br>↓Lactobacillaceae family,<br>↓Enterococcaceae family<br>↓Faecalibacterium prausnitzii species<br>**Actinobacteria phylum:**<br>↑Bifidobacterium genus<br>**Bacteroidetes phylum:**<br>↓Prevotellaceae family<br>**Verrucomicrobia phylum:**<br>↑Akkermansia muciniphila species<br>**Proteobacteria phylum:**<br>↑Enterobacteriaceae family<br><br>**PD with COMT inhibitor:**<br><br>↓**Firmicutes phylum:**<br>↓Faecalibacterium prausnitzii species
Bedarf et al.<br>2017	L-Dopa naïve PD patients had significant altered GI microbial composition in comparison to healthy controls.<br><br>Predictive functional analysis of L-Dopa naïve PD patients indicated differences in regulation of B-glucoronate and Tryptophan degradation pathways.	Case-Control study<br><br>PD (N=31)<br><br>Random Healthy Controls (N=28)	Metagenomics shotgun sequencing<br><br>Fecal samples	**L-Dopa naïve PD patients:**<br><br>**Firmicutes phylum:**<br>↓Erysipelotrichaceae family,<br>↓Eubacterium genus,<br>↓Clostridium genus,<br>↓Eubacterium biforme species,<br>↓Clostridium saccarolyticum species<br>**Verrucomicrobia phylum:**<br>↑Verrucomicrobiaceae family,<br>↑Akkermansia genus,<br>↑Akkermansia muciniphila species,<br>**Bacteroidetes phylum:**<br>↓Prevotellaceae family,<br>↓Prevotella genus, ↑Alistipes genus,<br>↑Alistipes shahii species,<br>↓Prevotella copri species

Hill-Burns et al. 2017	In comparison to healthy controls, PD patients were found to have significant altered GI microbial community.<br><br>Ruminococcaceae family were found to be associated with disease duration (>10 years).<br><br>Predictive functional analysis indicated 17 upregulated KEGG pathways and 9 downregulated pathways, including xenobiotics degradation and metabolism of plant-derived compounds.	Case-Control study<br><br>Total PD (N=197):<br>Random PD (N=143)<br>Spousal PD (N=54)<br><br>Total HC (N=130):<br>Random Healthy Controls (N=76),<br>Spousal Healthy Controls (N=54)	16S rRNA gene amplicon sequencing<br><br>Fecal samples	**<u>Non-medicated PD:</u>**<br><br>**Actinobacteria phylum:**<br>↑Bifidobacteriaceae family<br>↑Bifidobacterium OTU,<br>↑Bifidobacterium genus<br>**Bacteroidetes phylum:**<br>↑Parabacteroides OTU,<br>↑Prevotella OTU<br>**Firmicutes phylum:**<br>↑Lactobacillaceae family,<br>↑Tissierellaceae family,<br>↑Christensenellaceae family,<br>↓Lachnospiraceae family,<br>↓Blautia OTU, ↓Coprococcus OTU,<br>↓Roseburia OTU,<br>↓Faecalibacterium OTU,<br>↑Lactobacillus genus, ↓Blautia genus,<br>↓Roseburia genus,<br>↓Faecalibacterium genus<br>**Verrucomicrobia phylum:**<br>↑Verrucomicrobiaceae family,<br>↑Akkermansia genus<br>**Proteobacteria phylum:**<br>↓Pasteurellaceae family<br><br>**<u>Medicated PD:</u>**<br><br>**Actinobacteria phylum:**<br>↑Bifidobacteriaceae family<br>↑Bifidobacterium OTU,<br>↑Bifidobacterium genus,<br>**Bacteroidetes phylum:**<br>↑Prevotella OTU<br>**Firmicutes phylum:**<br>↑Lactobacillaceae family,<br>↑Tissierellaceae family,<br>↑Christensenellaceae family,<br>↓Lachnospiraceae family,<br>↓Blautia OTU ↓Coprococcus OTU,<br>↓Roseburia OTU,<br>↓Faecalibacterium OTU,<br>↑Lactobacillus genus, ↓Blautia genus,<br>↓Roseburia genus,<br>↓Faecalibacterium genus<br>**Verrucomicrobia phylum:**<br>↑Verrucomicrobiaceae family,<br>↑Akkermansia genus<br>**Proteobacteria phylum**:<br>↓Pasteurellaceae family
Hopfner et al. 2017	In comparison to healthy controls, PD participants were found to have significantly altered GI microbial community, in specifically a high abundance of Lactobacillaceae family, Enterococcaceae family, and Barnesiellaceae family.	Case-Control Study<br><br>PD (N=29)<br><br>Random Healthy Controls (N=29)	16S rRNA V1-V2 gene amplicon sequencing<br><br>Fecal samples	**Firmicutes phylum:**<br>↑Lactobacillaceae family,<br>↑Enterococcaceae family<br>**Bacteroidetes phylum:**<br>↑Barnesiellaceae family

Li et al. 2017	GI microbial composition was significantly altered in PD participants compared to healthy controls.<br><br>Faecalibacterium genus was found to be significantly lower, while Megasphaera genus was significantly higher in severe PD cohort.<br><br>Greater PD duration was associated with an increase in Proteus genus, Enterococcus genus, Escherichia-Shigella genus, Megasphaera genus, all of which are putative pathobionts. Greater PD duration was associated with a decrease in Blautia genus, Ruminococcus genus, Sporobacter genus and Haemophilus genus, all of which are cellulose degraders.	Case-Control study<br><br>PD (N=24)<br><br>Random Healthy Controls (N=14)	16S rRNA V3-V5 gene amplicon sequencing<br><br>Fecal samples	**Actinobacteria phylum:** ↑Coriobacteriaceae family<br>**Firmicutes phylum:** ↑Bacilli class, ↑Negativicutes class, ↑Veillonellaceae family, ↑Erysipelotrichaceae family, ↑Enterococcaceae family, ↑Acidaminococcus genus, ↑Enterococcus genus, ↑Megamonas genus, ↑Megasphaera genus, ↑Streptococcus genus, ↓Ruminococcus family, ↓Lachnospiraceae family, ↓Blautia genus, ↓Faecalibacterium genus, ↓Ruminococcus genus<br>**Proteobacteria phylum:** ↑Enterobacteriaceae family, ↑Moraxellaceae family, ↑Acinetobacter genus, ↑Escherichia-shigella genus, ↑Proteus genus<br>**Bacteroidetes phylum:** ↓Prevotellaceae family
Petrov et al. 2017	Patients with PD demonstrated significantly altered GI microbial composition when compared to healthy controls, causing changes in 9 genera and 15 species.	Case-Control study<br><br>PD (N=89)<br><br>Random Healthy Controls (N=66)	16S rRNA V3-V4 gene amplicon sequencing<br><br>Fecal samples	**Firmicutes phylum:** ↑Christensenella genus, ↑Lactobacillus genus, ↑Oscillospira genus, ↑Catabacter genus, ↓Dorea genus, ↓Faecalibacterium genus, ↑Christensenella minuta species, ↑Catabacter hongkongenesis species, ↑Lactobacillus mucosae species, ↑Ruminococcus bromii species, ↑Papillibacter cinnamivorans species, ↓Stoquefichus massiliensis species, ↓Blautia glucerasea species, ↓Dorea longicatena species, ↓Coprococcus eutactus species, ↓Ruminococcus callidus species<br><br>**Actinobacteria phylum:** ↑Bifidobacterium genus<br><br>**Bacteroidetes phylum:** ↓Bacteroides genus, ↓Prevotella genus, ↓Bacteroides massiliensis species, ↓Bacteroides coprocola species, ↓Bacteroides dorei species, ↓Bacteroides plebeus species, ↓Prevotella copri species

Pereira et al. 2017	Oral swab microbiota composition was significantly altered in PD patients in comparison to healthy controls.<br><br>Within the oral microbiota of PD patients, there were a high abundance of opportunistic pathogens.<br><br>Nasal swab microbiota in PD patients demonstrated differences in abundances of bacterial taxa, however lacked strong significance.	Case-Control study<br><br>Oral: PD (N=72)<br><br>Random Healthy Controls (N=76)<br><br>Nasal: PD (N=69)<br><br>Random Healthy Controls (N=67)	16S rRNA V3-V4 gene amplicon sequencing<br><br>Nasal Swab and Oral Swab samples	**<u>Oral swab microbiota in PD:</u>**<br><br>**Bacteroidetes phylum:**<br>↑Prevotellaceae family,<br>↑Prevotella genus,<br>↓Capnocytophaga genus,<br>↓unclassified Flavobacteriaceae OTU<br>**Firmicutes phylum:**<br>↑Veillonellaceae family,<br>↑Lactobacillaceae family,<br>↑Erysipelotrichaceae family,<br>↓Carnobacteriaceae family,<br>↑Veillonella genus,<br>↑Solobacterium genus,<br>↑Moryella genus,<br>↓Gemella genus,<br>**Fusobacteria phylum:**<br>↓Leptotrichiaceae family,<br>↓Leptotrichia genus,<br>**Proteobacteria phylum:**<br>↓Nisseriaceae family,<br>↓Pasterurellaceae family,<br>↓Micrococcaceae family,<br>↓Corynebacteriaceae family,<br>↓Kingella genus,<br>↓Haemophilus genus,<br>↓Neisseria genus,<br>↓Granulicatella genus<br>**Actinobacteria phylum:**<br>↑Coriobacteriaceae family,<br>↓Rothia genus, ↓Actinomyces genus,<br>↓Corynebacterium genus,<br><br>**<u>Nasal swab microbiota in PD:</u>**<br><br>**Firmicutes phylum:**<br>↑Staphylococcus OTU<br>**Actinobacteria phylum:**<br>↑Marmoricola genus<br>**Bacteroidetes phylum:**<br>↑Flavobacteriaceae family
Minato et al. 2017	Fecal samples were obtained from year 1 and year 2 from deteriorated and stable PD cohorts.<br><br>In comparison to year 1, deteriorated and stable PD groups exhibited a significant alteration in gut microbial communities in comparison to healthy controls.<br><br>When compared to stable PD group, deteriorated PD cohort demonstrated a significantly differentiated bacterial community. The change in bacterial communities between both cohorts, across 2 years, further correlates with a progressive PD pathology.	2-year Longitudinal study<br><br>PD (N=36): deteriorated PD (N=18), stable PD (N=18)<br><br>No controls	Quantitative PCR of 16S and 23S rRNA<br><br>Fecal samples	**<u>Deteriorated versus stable PD – year 1:</u>**<br><br>**Deteriorated PD:**<br>**Actinobacteria phylum:**<br>↓Bifidobacterium genus<br><br>**<u>Deteriorated versus stable PD - year 2:</u>**<br><br>**Deteriorated PD**<br>**Firmicutes phylum:**<br>↓Lactobacillus gasseri species<br>**Bacteroidetes phylum:**<br>↓Prevotella genus<br><br>**Stable PD:**<br>**Actinobacteria phylum:**<br>↓Bifidobacterium genus,<br>↓Aptobium cluster<br>**Firmicutes phylum:**<br>↓Enterococcus genus,<br>↓Lactobacillus gasseri species,<br>↓Lactobacillis reuteri species,<br>↓Clostridium leptum species<br>**Bacteroidetes phylum:**<br>↓Prevotella genus,<br>↓Bacteroides fragilis species

Heintz-Buschart et al. 2018	In comparison to healthy controls, PD patients had significant altered GI microbial composition, but lacked a strong significant alteration in nasal microbiome.<br><br>Within the PD patients, Anaerotruncus genus, Clostridium genus, and Lachnospiraceae family were found to be related to motor symptoms. Anaerotruncus genus and Akkermansia genus were found to be related to nonmotor symptoms.	Case-Control study<br><br>PD (N=76)<br><br>Random Healthy Controls (N=78)	16S and 18S rRNA V4 gene amplicon sequencing, metagenomic shotgun sequencing<br><br>16S rRNA: Nasal Wash (N=147) and Fecal (N=84) samples<br><br>18S rRNA: 61 Fecal samples	**Fecal PD:**<br><br>**Firmicutes phylum:**<br>↑Acidaminococcaceae class,<br>↑Erysipelotrichaceae order,<br>↑Ruminococcaceae family,<br>↑Clostridiales family,<br>↑Lachnospiraceae family,<br>↑Flavonifractor genus,<br>↑Ruminococcus genus,<br>↑Megasphaera genus,<br>↑Mitsuokella genus,<br>↑Clostridium XVIa genus,<br>↑Anaerotruncus genus<br>**Verrucomicrobia phylum:**<br>↑Verrucomicrobiales order,<br>↑Verrucomicrobiae class,<br>↑Verrucomicrobiaceae family,<br>↑Akkermansia genus,<br>**Actinobacteria phylum:**<br>↑Olsenella genus<br>**Proteobacteria phylum:**<br>↑Sutterella genus<br>**Bacteroidetes phylum:**<br>↑Prevotellaceae family,<br>↑Prevotella genus,<br>↑Bacteroides genus,<br>↑Butyricimonas genus<br><br>**Nasal fluid of PD (taxa trending toward significance):**<br><br>**Firmicutes phylum:**<br>↑Listeriaceae family,<br>↑Bacillaceae family,<br>↑Ruminococcaceae family,<br>↑Carnobacteriaceae family<br>**Actinobacteria phylum:**<br>↑Micrococcaceae family<br>**Proteobacteria phylum:**<br>↑Hydrogenophilaceae family,<br>↑Hyphomicrobiaceae family,<br>↑Desulfovibrionaceae family
Lin et al. 2018	Patients with PD exhibited significant altered abundance of GI microbial community in comparison to healthy controls.	Case-Control study<br><br>PD (N=75)<br><br>Spousal Healthy Controls (N=45)	16s rRNA V4 gene amplicon sequencing<br><br>Fecal samples	**Firmicutes phylum:**<br>↑Eubacteruaceae family,<br>↑Aerococcaceae family,<br>↓Lachnospiraceae family,<br>↓Streptococcaceae family,<br>↓Gemellaceae family<br>**Actinobacteria phylum:**<br>↑Bifidobacteriaceae family,<br>↓Actinomycetaceae family,<br>↓Micrococcaceae family,<br>↓Intrasporangeaceae family,<br>↓Brevibacteriaceae family<br>**Proteobacteria phylum:**<br>↑Desulfovibrioaceae family,<br>↓Pasterurellaceae family,<br>↓Methylobacteriaceae family,<br>↓Comamonadaceae family,<br>↓Halomonadaceae family,<br>↓Hyphomonadaceae family,<br>↓Brucellaceae family,<br>↓Xanthomonadaceae family,<br>↓Sphingomonadaceae family,<br>↓Idiomarinaceae family<br>**Euryarchaeota phylum:**<br>↓Methanobacteriaceae family

Qian et al. 2018	Patients with PD exhibited significant altered GI microbial composition when compared to healthy controls.<br><br>Further disease duration was associated with a reduction in Esherichia/Shigella genus.<br><br>Predictive functional analysis demonstrated pathways involving metabolism of cofactors and vitamins, porphyrin and chlorophyll metabolism, and biotin metabolism were less abundant in PD fecal samples. However, pathways involving energy metabolism, flavone and flavonal biosynthesis, fatty acid biosynthesis and apoptosis were more abundant in PD fecal samples.	Case-Control study<br><br>PD (N=45)<br><br>Spousal Healthy Controls (N=45)	16s rRNA V3-V4 gene amplicon sequencing<br><br>Fecal Samples	**PD:**<br><br>**Proteobacteria phylum:**<br>↑Sphingomonas genus,<br>↑Aquabacterium genus<br>**Firmicutes phylum:**<br>↑Clostridium IV genus,<br>↑Clostridium XVIII genus,<br>↑Butyricicoccus genus,<br>↑Holdemania genus,<br>↑Anaerotruncus genus,<br>↓Lactobacillus genus<br>**Bacteroidetes phylum:**<br>↓Sediminibacterium genus<br><br>**PD with Levodopa Equivalent Doses:**<br><br>**Firmicutes phylum:** ↓Dorea genus,<br>↓Phascolarctobacterium genus
Barichella et al. 2019	In comparison to healthy controls, PD patients demonstrated significantly altered GI microbial community, specifically a reduction in Lachnospiraceae and an increase in Lactobacillaecea and Christensenellaceae. Such alterations are associated with increased severity in PD.<br><br>Predictive functional analysis revealed 11 upregulated KEGG pathways and 15 downregulated KEGG pathways in de novo PD patients, when compared to healthy controls. Many of the altered pathways are involved in cellular processes, human diseases, metabolism, and more.	Case-Control study<br><br>PD (N=193)<br><br>Random Healthy Controls (N=113)	16S rRNA V3-V4 gene amplicon sequencing<br><br>Fecal samples	**PD:**<br><br>**Verrucomicrobia phylum:**<br>↑Verrucomicrobiaceae family,<br>**↑Akkermansia genus**<br>**Proteobacteria phylum:**<br>↑Enterobacteriaceae family<br>**Firmicutes phylum:**<br>↑Christensenellaceae family,<br>↑Lactobacillaceae family,<br>↓Lachnospiraceae family,<br>↑Ruminococcus genus,<br>↑Oscillospira genus,<br>↓Roseburia genus<br>**Actinobacteria phylum:**<br>↑Coriobacteriaceae family,<br>↑Bifidobacteriaceae family<br>**Bacteroidetes phylum:**<br>↑Parabacteroides genus<br><br>**PD with COMT inhibitors:**<br><br>**Bacteroidetes phylum:**<br>↑Porphyromonadaceae family<br>**↑Proteobacteria phylum**<br>**Firmicutes phylum:**<br>↑Lactobacillaceae family,<br>↓Lachnospiraceae family,<br>↓Ruminococcaceae family<br>**↑Actinobacteria phylum**<br><br>**PD with Proton Pump Inhibitors:**<br><br>**Firmicutes phylum:**<br>↑Christensenellaceae family,<br>↑Lactobacillaceae family

Aho et al. 2019	Gut microbial compositions exhibited significant alterations in PD patients when compared to healthy controls.<br><br>A two-year follow-up demonstrated a significant alteration in microbial communities in PD patients in comparison to healthy control.	Longitudinal study (follow up (2-2.5 year) Scheperjans 2015)<br><br>PD (N=64)<br><br>Random Healthy Controls (N=64)	16S rRNA V3-V4 gene amplicon sequencing<br><br>Fecal samples	**Baseline PD:**<br><br>**Bacteroidetes phylum:**<br>↑Rikenellaceae family,<br>↑OTU 0300 Alistipes,<br>↑OTU 0098 Bacteroides<br>**Firmicutes phylum:**<br>↑OTU 0513 Anaerotruncus,<br>↓Lachnospiraceae family,<br>↓Roseburia genus, ↓Blautia genus,<br>↓Clostridium XIVa genus,<br>↓Roseburia OTU, ↓Blautia OTU<br>**Verrucomicrobia phylum:**<br>↓Puniceicoccaceae family<br><br>**2nd Year Follow up in PD:**<br><br>**Actinobacteria phylum:**<br>↑Bifidobacteriaceae family,<br>↑Bifidobacterium genus<br>**Firmicutes phylum:**<br>↑Lactobacillaceae family,<br>↑Lactobacillus genus,<br>↓Roseburia genus,<br>↓Clostridium XIVa genus,<br>↓Roseburia OTU, ↓Blautia OTU,<br>↓Ruminococcus OTU<br>**Bacteroidetes phylum:**<br>↓Prevotellaceae family,<br>↑OTU 0379 Alstipes,<br>↑ OTU 0464 Lactobacillus,<br>↓Prevotella genus, ↓Bacteroides OTU<br>**Verrucomicrobia phylum:**<br>↓Puniceicoccaceae family

Li et al.<br>2019	PD patients showed significant altered gut microbial taxa in comparison to healthy controls.<br><br>PD patients also demonstrated a decrease in species richness and phylogenetic diversity in comparison to healthy controls.	Case-Control study<br><br>PD (N=51)<br><br>Healthy Controls (N= 48)<br>Spousal Healthy Controls (N=39)<br>Random Healthy Controls (N=9)	16S rRNA V4 gene amplicon sequencing<br><br>Fecal samples	**Verrucomicrobia phylum:**<br>↑Verrucomicrobiaceae family,<br>↑Verrucomicrobiales order,<br>↑Akkermansia genus<br>**Firmicutes phylum:**<br>↑Clostridia class,<br>↑Negativicutes order,<br>↑Clostridiales order,<br>↑Selenomonadales order,<br>↑Ruminococcaceae family,<br>↑Veillonellaceae family,<br>↑Acidaminococcaceae family,<br>↑Ruminococcus genus,<br>↑Eubacterium coprostanoligenes group,<br>↑Coprococcus genus,<br>↑Phascolarctobacterium genus,<br>↑Roseburia genus,<br>↑Lachnospiraceae family,<br>↑Ruminococcus callidus species,<br>↑Roseburia inulinivorans species,<br>↑Ruminococcus torques species,<br>↓Lactobacillus genus, ↓ Bacilli class,<br>↓Lactobacillales order,<br>↓Lactobacillaceae family,<br>↓Streptococcus genus,<br>↓Eubacterium halii species<br>**Eurarchaeota phylum:**<br>↑Methanobrevibacter genus,<br>↑Methanobrevibacter smithii species<br>**Bacteroidetes phylum:**<br>↑Porphyromonadaceae family,<br>↑Rikenellaceae family,<br>↑Parabacteroides genus,<br>↑Alstipes genus,<br>↑Parabacteroides merdae species,<br>↑Prevotella copri species
